# Association of serum 25-hydroxyvitamin D status with bone mineral density in 0-7 year old children

**DOI:** 10.18632/oncotarget.13097

**Published:** 2016-11-04

**Authors:** Yanrong Fu, Youfang Hu, Zhenying Qin, Yan Zhao, Zi Yang, Yinfang Li, Guanyu Liang, Heyun Lv, Hong Hong, Yuan Song, Yarong Wei, Hongni Yue, Wen Zheng, Guoqin Liu, Yufei Ni, Mei Zhu, Aiping Wu, Juhua Yan, Chenbo Ji, Xirong Guo, Juan Wen, Rui Qin

**Affiliations:** ^1^ Department of Child Health Care, the Second Affiliated Hospital of Nanjing Medical University, Nanjing Medical University, Nanjing, 210011, China; ^2^ Department of Child Health Care, Jiangsu Women and Children Health Hospital, Women and Child Branch Hospital of Jiangsu Province Hospital, the First Affiliated Hospital with Nanjing Medical University, Nanjing, 210036, China; ^3^ Nanjing Maternity and Child Health Care Institute, Nanjing Maternity and Child Health Care Hospital, Obstetrics and Gynecology Hospital Affiliated to Nanjing Medical University, Nanjing, 210029, China; ^4^ Department of Pediatrics, Nanjing First Hospital, Nanjing Medical University, Nanjing, 210006, China; ^5^ Department of Child Health Care, Jiangning Maternity and Child Health Care Institute, Nanjing, 211100, China; ^6^ Department of Child Health Care, Drum Tower Maternity and Child Health Care Institute, Nanjing, 210009, China; ^7^ Department of Child Health Care, Suzhou Municipal Hospital, Suzhou, 215000, China; ^8^ Department of Child Health Care, Wuxi Maternity and Child Health Care Hospital, Wuxi, 214000, China; ^9^ Department of Child Health Care, Huai'an Maternity and Child Health Care Hospital, Huai'an, 223001, China; ^10^ Department of Child Health Care, Yancheng Maternity and Child Health Care Institute, Yancheng, 224000, China; ^11^ Department of Child Health Care, Dafeng Maternity and Child Health Care Hospital, Dafeng, 224100, China; ^12^ Department of Child Health Care, Nantong Maternity and Child Health Care Hospital, Nantong, 226000, China; ^13^ Department of Child Health Care, Xuzhou Children's Hospital, Xuzhou, 221000, China; ^14^ Department of Child Health Care, Xinghua Maternity and Child Health Care Hospital, Xinghua, 225700, China; ^15^ Department of Child Health Care, Kunshan Maternity and Child Health Care Institute, Kunshan, 215300, China

**Keywords:** serum 25-hydoxyvitamin D, bone mineral density, 0-7 year old children

## Abstract

**Objective:**

To describe the status of serum 25-hydoxyvitamin D [25(OH)D] concentrations and identify the relationship between 25(OH)D and bone mineral density (BMD). In an effort to explore the appropriate definition of vitamin D (VD) deficiency in 0–7 year old children.

**Results:**

The median serum 25(OH)D concentrations was 62.9 nmol/L and 28.9% of the children had a low 25(OH)D (< 50 nmol/L). And a linear relation between 25(OH)D concentrations and BMD was surveyed (r = 0.144 , *P* < 0.001). After adjusting for the confounders, serum 25(OH)D was positively associated with BMD (β = 172.0, 95%CI = 142.8–201.2, *P* < 0.001), and low 25(OH)D (< 75 nmol/L) had a high stake for low BMD (OR = 1.424, 95%CI = 1.145–1.769, *P* = 0.001). Additionally, there was a nonlinear relation between 25(OH)D and low BMD, and a critical value for 25(OH)D of 75 nmol/L appeared for low BMD. The prevalence of low BMD was 14.1% in children with 25(OH)D ≥ 75 nmol/L, much lower than that of the concentrations between 50–75 nmol/L and < 50 nmol/L.

**Materials and Methods:**

A total of 4,846 children 0–7 years old were recruited in Jiangsu Province, China. BMD and serum 25(OH)D concentrations were determined by quantitative ultrasound and enzyme-linked immunosorbent assay, respectively. Linear regression and logistic regression analyses were used to assess the association of 25(OH)D concentrations with BMD.

**Conclusions:**

Serum 25(OH)D concentrations was related with BMD and 25(OH)D concentrations < 75 nmol/L might be a more appropriate definition of VD deficiency in 0–7 year old children.

## INTRODUCTION

Vitamin D (VD) deficiency is common among children due to the combined effects of additional growth needs, inadequate intake and limited sunlight exposure, which has become an grave public health issue in developed and developing countries [1]. Nowadays VD deficiency is severe among children in China, with a reported prevalence of 30%–70% in the north [2] and 10%–40% in the south of China [3, 4]. One of the classic roles of VD is to maintain the homeostasis of calcium and phosphorus by absorption in the intestines and mobilization from the skeleton [5, 6]. Since the 1920s, VD was identified and its deficiency was linked to rickets [7] and more and more studies showed that VD deficiency was associated with low BMD [8–11]. With the extensive researches and global emphasis on the importance of supplement, nearly 30 years ago, the appearance of serious nutritional rickets was considered to vanish [12], however, low BMD remained prevalent [8, 10, 13]. On the basis of the international society for Clinical Densitometry States, low BMD can be one of the indicators to diagnose osteoporosis and low bone mass [14]. Therefore it is essential to maintain sustained circulating VD and bone mass from childhood to avoid these ricks.

25(OH)D is the storage form of VD, and serum 25(OH)D concentrations is an indicator of VD status. There is a lack of consensus on definition regarding VD deficiency in young children. It is supported by the Institute of Medicine (IOM) and the Endocrine Society's practice guidelines that VD deficiency considering bone health is defined as the serum free 25(OH)D concentrations < 50 nmol/L, while the VD insufficiency is defined as the 25(OH)D concentrations of 52–72 nmol/L [15, 16]. A study by Vieth et al. suggested that the maximal inhibition of parathyroid hormone (PTH) by circulating 25(OH)D occurred at the concentrations of 25(OH)D > 80 nmol/L [17]. Some studies including our previous studies supported that when the concentrations > 75 nmol/L, the PTH levels plateaued [18]. But in late 2010, the IOM reported another statement that the concentrations of 25(OH)D > 50 nmol/L was sufficient for bone health. Whether the definition of deficiency should be extent to 75 nmol/L or greater is less clear [7].

Although the serum free 25(OH)D concentrations can partly reflect the status of VD, in the clinic examination, BMD is a vital functional outcome of VD deficiency in children [4, 19]. Quantitative ultrasound measure techniques are used wildly as an effective and convenient tool to measure BMD, particularly in the primary hospitals of China. According to the previous study, the measurement of speed of sound (SOS; m/s) is the common indicator of BMD [20].

At present, the relation between serum 25(OH)D concentrations and BMD in children remains confusion [21]. This study was designed to describe the status of 25(OH)D serum concentrations and determine the relation between serum 25(OH)D concentrations and BMD in 0–7 year old children, and to provide certain reference for the definition of VD deficiency of Chinese children.

## RESULTS

A total of 4,846 serum samples were tested for 25(OH)D, with linked information related to the bone mineral density available for 4,622 (95.4%). We excluded 90 children who had liver, kidney or cardiovascular diseases, or who had 25(OH)D concentrations outside the assay detection limits. At last, a total of 4,532 (2380 boys and 2152 girls) children aged from 1 months to 7 years were included in the analysis. The mean (SD) age of children was 35.8 (20.9) months. 5.2% of the children were preterm infants (gestational age < 37 weeks), while the mean (SD) birth weight was 3.4 (0.5) kg. The percentile distributions of 25(OH)D concentrations and selected characteristics are presented in Table 1. The median concentrations was 62.9 nmol/L and 28.9% of the children had a low 25(OH)D (< 50 nmol/L). The serum free 25(OH)D concentrations was associated with gender, age, gestational season, milk intake during pregnancy and for children, VD or calcium supplementation during pregnancy and for children, and time of outdoor activity during pregnancy and for children (*P* < 0.05). With the increasing quartile of 25(OH)D, the BMD increased (*P* < 0.001). The lowest quartile of 25(OH)D had a highest prevalence (25.9%) of low BMD (*P* < 0.001). Compared with children with 25(OH)D > 50 nmol/L, children with low 25(OH)D (< 50 nmol/L) had a higher prevalence of low BMD (*P* < 0.001). BMD was also associated with age, gestational age and season, delivery mode, and time of outdoor activity during pregnancy and for children (*P* < 0.05). In addition, gestational season in autumn or winter, spontaneous delivery, milk intake for children < 250 ml/day, no VD or calcium supplementation during pregnancy and for children, and time of outdoor activity during pregnancy and for children < 2 h all increased the stake of low BMD (*P* < 0.05).

**Table 1:** Effects of risk factors on 25(OH)D and BMD

Variables	N (%)	25(OH)D		BMD		Low BMD	
		Mean ± SD	*P*	Mean ± SD	*P*	N (%)	*P*
25(OH)D quartiles (nmol/L)							
≤ 47.7	1134 (25.0)	34.5 ± 9.8	< 0.001	3336.3 ± 230.7	< 0.001	294 (25.9)	< 0.001
47.8 - 62.9	1136 (25.1)	55.1 ± 4.4		3404.9 ± 230.7		212 (18.7)	
63.0 - 78.0	1138 (25.1)	70.2 ± 4.3		3383.7 ± 238.3		204 (17.9)	
≥ 78.1	1124 (24.8)	95.5 ± 16.6		3399.8 ± 225.5		141 (12.5)	
25(OH)D concentrations							
< 50 nmol/L	1310 (28.9)	36.4 ± 10.3	< 0.001	3345.8 ± 231.7	< 0.001	335 (25.6)	< 0.001
50–75 nmol/L	1864 (41.1)	62.2 ± 7.1		3397.7 ± 233.8		325 (17.4)	
≥ 75 nmol/L	1358 (30.0)	92.2 ± 16.7		3392.5 ± 229.1		191 (14.1)	
Gender							
Male	2380 (52.5)	65.2 ± 24.6	< 0.001	3386.8 ± 236.2	0.087	444 (18.7)	0.825
Female	2152 (47.5)	62.2 ± 24.1		3374.9 ± 229.0		407 (18.9)	
Age							
0–6 months	413 (9.1)	59.7 ± 23.9	< 0.001	2991.0 ± 149.6	< 0.001	65 (15.7)	0.379
7–12 months	436 (9.6)	65.2 ± 25.1		3149.5 ± 124.2		73 (16.7)	
13–24 months	804 (17.7)	66.9 ± 24.1		3298.3 ± 170.4		143 (17.8)	
25–36 months	696 (15.4)	66.6 ± 24.2		3400.7 ± 182.0		134 (19.3)	
37–48 months	738 (16.3)	65.1 ± 24.2		3499.8 ± 145.8		146 (19.8)	
49–60 months	749 (16.5)	61.2 ± 24.5		3534.9 ± 133.9		149 (19.9)	
≥ 61 months	696 (15.4)	60.1 ± 23.9		3542.6 ± 151.9		141 (20.3)	
Gestational age							
Term infant	4143 (94.8)	63.7 ± 24.5	0.351	3379.9 ± 232.4	0.011	768 (18.5)	0.053
Preterm infant	228 (5.2)	62.2 ± 25.5		3334.6 ± 261.4		54 (23.7)	
Gestational season							
Spring	1346 (29.7)	60.6 ± 24.8	< 0.001	3447.3 ± 222.1	< 0.001	243 (18.1)	< 0.001
Summer	1810 (39.9)	63.6 ± 26.8		3334.9 ± 229.8		276 (15.2)	
Autumn	580 (12.8)	69.5 ± 21.8		3370.8 ± 239.5		139 (24.0)	
Winter	796 (17.6)	65.2 ± 18.3		3381.9 ± 226.6		193 (24.2)	
Delivery mode							
Spontaneous delivery	1913 (43.2)	63.6 ± 25.3	0.502	3369.9 ± 238.7	0.003	391 (20.4)	0.006
Cesarean delivery	2516 (56.8)	64.1 ± 23.8		3390.7 ± 227.2		432 (17.2)	
Birth weight							
< 2500 g	187 (4.2)	65.2 ± 24.3	0.609	3364.7 ± 235.7	0.346	32 (17.1)	0.787
2500–4000 g	3903 (87.3)	63.7 ± 24.6		3379.6 ± 232.9		733 (18.8)	
≥ 4000 g	379 (8.5)	63.0 ± 22.4		3393.7 ± 231.5		74 (19.5)	
Feeding patterns							
Breast feeding	2339 (52.0)	63.7 ± 24.8	0.179	3386.3 ± 233.5	0.073	441 (18.9)	0.128
Mixed feeding	1308 (29.1)	63.3 ± 22.7		3368.6 ± 233.0		259 (19.8)	
Artificial feeding	849 (18.9)	65.2 ± 25.6		3385.5 ± 231.0		139 (16.4)	
Milk intake during pregnancy							
≥ 250 ml/day	1378 (34.0)	65.0 ± 26.6	0.047	3366.1 ± 234.4	0.300	247 (17.9)	0.359
< 250 ml/day	2679 (66.0)	63.3 ± 23.4		3374.3 ± 239.9		512 (19.1)	
VD or calcium supplementation during pregnancy							
Yes	1633 (39.9)	64.8 ± 25.1	0.027	3380.7 ± 241.5	0.233	224 (13.7)	< 0.001
No	2464 (60.1)	63.0 ± 23.6		3371.5 ± 238.3		538 (21.8)	
Time of outdoor activity during pregnancy							
< 1 h	696 (17.7)	61.5 ± 24.6	< 0.001	3322.4 ± 247.1	< 0.001	174 (25.0)	< 0.001
1–2 h	1999 (51.0)	63.0 ± 23.9		3364.9 ± 234.4		387 (19.4)	
> 2 h	1227 (31.3)	66.3 ± 24.0		3388.5 ± 232.8		179 (14.6)	
Milk intake for children							
≥ 250 ml/day	2100 (53.8)	64.9 ± 23.9	0.018	3374.3 ± 222.6	0.200	336 (16.0)	< 0.001
< 250 ml/day	1804 (46.2)	63.1 ± 23.7		3384.1 ± 249.2		385 (21.3)	
VD or calcium supplementation for children							
Yes	1971 (50.4)	64.3 ± 24.4	0.025	3373.2 ± 249.9	0.684	291 (14.8)	< 0.001
No	1942 (49.6)	62.6 ± 23.9		3376.3 ± 228.2		457 (23.5)	
Time of outdoor activity for children							
< 1 h	435 (9.7)	59.6 ± 20.9	< 0.001	3231.7 ± 272.1	< 0.001	116 (26.7)	< 0.001
1–2 h	2014 (44.8)	62.8 ± 24.2		3364.2 ± 236.7		435 (21.6)	
> 2 h	2042 (45.5)	65.6 ± 25.0		3429.3 ± 202.6		290 (14.2)	

**Figure 1:**
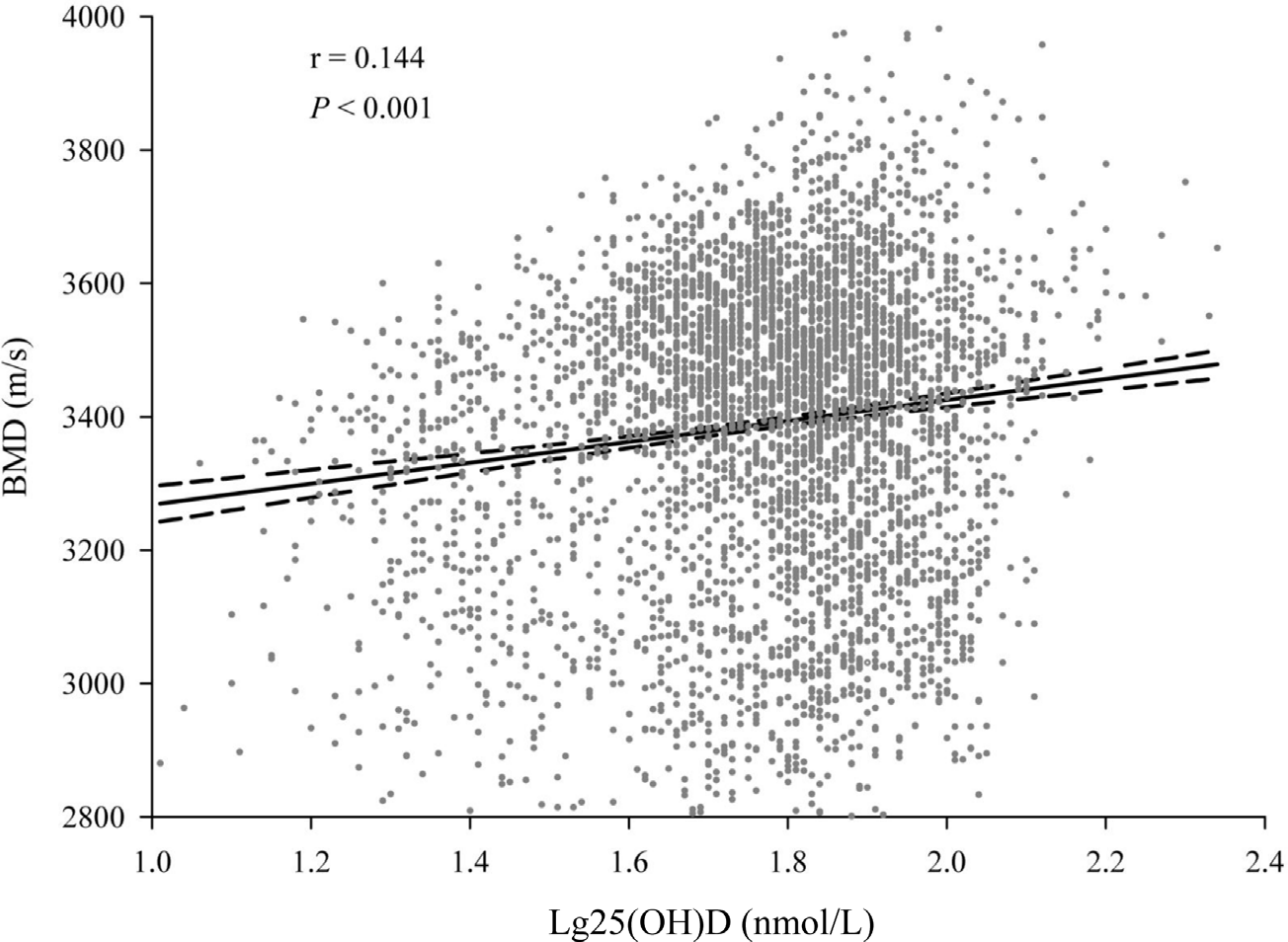
The relation between serum 25(OH)D concentrations and BMD (A) linear relation between 25(OH)D concentrations and BMD was observed. The correlation coefficient was 0.144 (*P* < 0.001).

As shown in Figure 1, a linear relation between the 25(OH)D concentrations and BMD was discovered (r = 0.144 , *P* < 0.001). After adjusting the confounders of gender, age, body mass index, gestational age and season, delivery mode, birth weight, feeding patterns, milk intake during pregnancy and for children, maternal and children's VD or calcium supplementation, and time of outdoor activity during pregnancy and for children, the 25(OH)D concentrations was positively related with BMD (β = 172.0, 95%CI = 142.8–201.2, *P* < 0.001). Compared with the children of 25(OH)D ≥ 75 nmol/L, low 25(OH)D (< 50 nmol/L) had a high stake of low BMD (OR = 1.648, 95%CI = 1.347–2.017, *P* < 0.001), and 25(OH)D < 75 nmol/L also had a significantly high stake for low BMD (OR = 1.424, 95%CI = 1.145–1.769, *P* = 0.001) (Table 2).

**Table 2:** Adjusted association between 25(OH)D and BMD

Variables	BMD^a^		Low BMD^a^	
	β (95% CI)	P	OR (95% CI)	P
Lg 25(OH)D	172.0 (142.8, 201.2)	< 0.001	0.197 (0.117, 0.331)	< 0.001
25(OH)D levels				
25(OH)D (≥ 75 nmol/L)	0.0 (ref)		1.000 (ref)	
Low 25(OH)D (< 50 nmol/L)	−52.3 (−64.0, −40.6)	< 0.001	1.648 (1.347, 2.017)	< 0.001
Low 25(OH)D (< 75 nmol/L)	−40.2 (−51.8, −28.7)	< 0.001	1.424 (1.145, 1.769)	0.001

a Adjusted: gender, age, body mass index, gestational age and season, delivery mode, birth weight, feeding patterns, milk intake during pregnancy and for children, VD or calcium supplementation during pregnancy and for children, and time of outdoor activity during pregnancy and for children.

**Figure 2:**
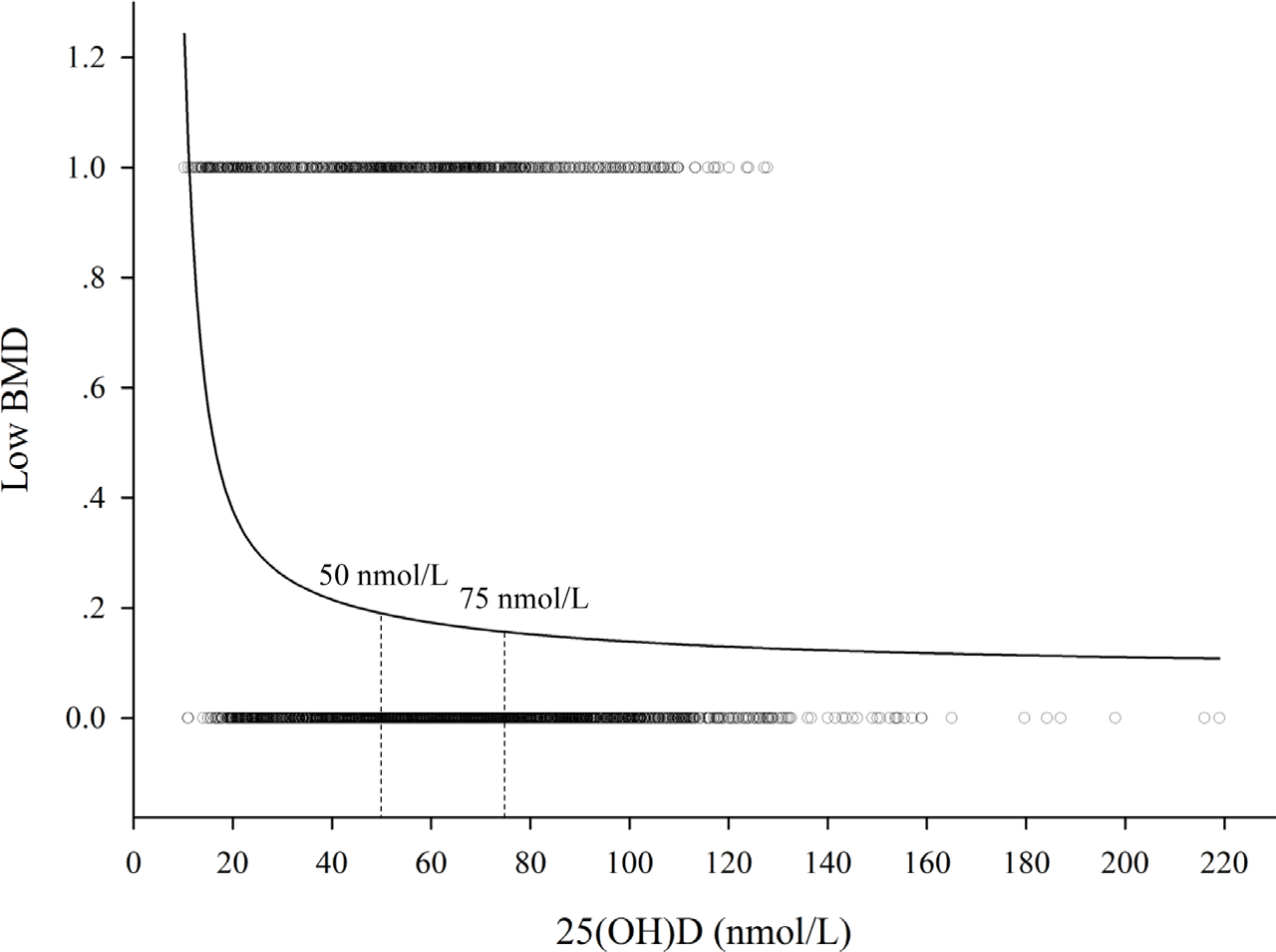
The relation between serum 25(OH)D concentrations and low BMD (A) nonlinear relation between the serum 25(OH)D and low BMD was observed. When the 25(OH)D concentrations reached 75 nmol/L, the prevalence of low BMD plateaued.

**Figure 3:**
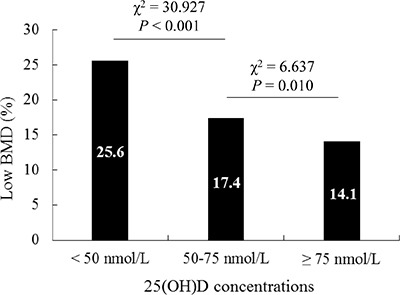
The effect of different classification of 25(OH)D and the prevalence of low BMD The prevalence of low BMD in children with 25(OH)D concentrations ≥ 75 nmol/L was significantly lower than that of the serum concentrations between 50–75 nmol/L and < 50 nmol/L (*P* < 0.05).

Then we conducted a stratified analysis on the association between 25(OH)D concentrations and the stake of low BMD (Table 3). When the concentrations was < 50 nmol/L, the stake of low BMD decreased along with the increase of 25(OH)D concentrations (OR = 0.982, 95%CI = 0.965–0.998, *P* = 0.031); While when the concentrations was < 75 nmol/L, the stake remain decreased (OR = 0.984, 95%CI = 0.977–0.991, *P* < 0.001). However, when 25(OH)D concentrations was ≥ 75 nmol/L, the association was not observed (*P* > 0.05). Consistently, there was a nonlinear relation between the serum 25(OH)D and low BMD. When the 25(OH)D concentration reached 75 nmol/L, the prevalence of low BMD plateaued (Figure 2). The prevalence of low BMD was 14.1% among children with 25(OH)D concentrations ≥ 75 nmol/L, which was significantly lower than 17.4% in children with the concentrations between 50–75 nmol/L and 25.6% in children with the concentrations < 50 nmol/L (*P* < 0.05) (Figure 3).

**Table 3:** Stratified analyses on association between 25(OH)D concentrations and low BMD

25(OH)D concentrations	OR (95% CI) ^a^	P ^a^
< 50 nmol/L	0.982 (0.965, 0.998)	0.031
< 75 nmol/L	0.984 (0.977, 0.991)	< 0.001
≥ 75 nmol/L	0.986 (0.971, 1.000)	0.052

a Adjusted: gender, age, body mass index, gestational age and season, delivery mode, birth weight, feeding patterns, milk intake during pregnancy and for children, VD or calcium supplementation during pregnancy and for children, and time of outdoor activity during pregnancy and for children.

## DISCUSSION

There had been lack of large epidemiological studies of VD situation in Chinese young children. Our study made up these shortcomings. As one of the biggest provinces of China, the state of VD deficiency of young children in Jiangsu Province allowed of no optimism. The study indicated that nearly 30% of 0–7 year old children had serum 25(OH)D concentrations under 50 nmol/L, while if we improved the definition standard to 75 nmol/L, almost 70% of whom had VD deficiency. The situation was more severe than that of Shanghai and Sichuan Province of China [4, 22]. The median 25(OH)D concentrations in this study was 62.9 nmol/L, which was much lower than that of US population based on the National Health and Nutrition Examination 2000–2004 survey (for 1–5 year old children, the mean serum 25(OH)D concentrations was 76.5 nmol/L) [23].

As is known to us, sunlight exposure plays an important role in serum VD concentrations. Considering the limited sunlight exposure nowadays, dietary intake of VD is necessary. It is suggested by the Endocrine Society's practice guidelines that children under 1 year old should take 400–1000 IU/d and then 600–1000 IU/d until 18 years old [16]. According to the newest suggestion of the Pediatric Branch of Chinese Medical Association, Chinese children are recommended to take 400–800 IU/d of VD after birth to 2 years old. And for premature infants, the supplements are 800–1000 IU/d after birth to 3 months then 400–800 IU/d until 2 years old. However, due to the misunderstanding of VD, many parents dared about the intoxication of extended taken of VD and did not follow the advice of doctors strictly. Early in the 1920s, surveys of VD toxicity appeared. In fact, these children who died had received nearly 20,000,000 units of VD, which were pharmacological doses and not within the physiological range [24]. Nowadays, it was confirmed by many researchers including Vieth et al. that supplement of 10,000 IU/d to 5 months and longer was safe and without adverse events [25]. While another research involving divers without sunlight exposure for 6 months showed that even 600 IU/d VD could not maintain the circulating serum 25(OH)D concentrations [24]. Unfortunately, it was not clearly that what dosages of VD were physiologic and what were pharmacologic. Because of the misunderstandings, fear of causing VD intoxication in children has continued up to now. That is why the medical scientists are reluctant to improve vitamin D requirements and the standard of VD deficiency.

For the serum 25(OH)D measurement, the liquid chromatography-tandem mass spectrometry (LC-MS/MS) method is generally considered to be the gold standard. However, the enzyme immunoassay was used in our study. Although the immunoassay may result in a little negative biases and misclassification of participants for vitamin D sufficiency when compared with the LC-MS/MS assay [26], it is more readily available and require small volume sampling, ideal for testing [27]. For assessment of BMD, the dual energy X-ray absorptiometry is the best way. However, it has only been used in major hospitals for its expensive and limited usage. Whereas quantitative ultrasound is of low cost, convenient and lack of radiation, which is used wildly to reflect the BMD of young children especially in clinical routine health exams [3, 4]. The study involving the relation between quantitative ultrasound BMD and serum 25(OH)D were relatively rare, especially in China. A study of small sample size of 203 children in Shanghai, China supported that BMD increased with the increase of the 25(OH)D concentrations, and suggested a threshold for the 25(OH)D concentrations of 20 ng/ml existed for low BMD [4]. Another study of 6,838 children in Sichuan province of China reported that VD concentrations might be correlated with BMD within a certain range (serum 25(OH)D concentrations between 50–75 nmol/L) [22]. The limitations of the above studies were lack of comprehensive design with large scale and multi-center or without adjusting for confounding factors. In this study, we conducted a multi-center case-control study, and taken into account the potential confounding factors. It was confirmed in our study along with the fact that low BMD of children was possibly to decrease when the concentrations was over 50 nmol/L. We provided a certain reference for the definition of VD deficiency that 25(OH)D concentrations under 75 nmol/L might be more appropriate than the concentrations under 50 nmol/L in 0–7 year old children. According to the report by Heaney et al., the calcium absorption of intestines was reduced in those who exhibit serum 25(OH)D concentrations of 50 nmol/L compared to the subjects with 25(OH)D concentrations more than 80 nmol/L [28]. However, more evidence is needed to support the definition of VD deficiency in young Chinese children, extending to 75 or even 80 nmol/L. Of course, our study had several shortcomings that should be noted. This study just focused on 13 Child Health Care centers of Jiangsu Province, which did not include other provinces of China. Further studies incorporating diverse populations and long term effect of VD deficiency are warranted to validate and extend the findings.

## MATERIALS AND METHODS

The study was approved by the institutional review board of the Jiangsu Women and Children Health Hospital, the First Affiliated Hospital with Nanjing Medical University. The methods were carried out according to the approved guidelines and conformed to the Declaration of Helsinki. All the participants or the guardians signed informed consent.

### Participants and study design

A nested case-control study was conducted on the basis of a study population of more than 50,000 children (0–7 year old) who were attending health check-up between April 2014 and March 2015 in 13 Children's Health Care Centers of Jiangsu Province (between 30 and 36 degrees north latitude), China. The children with metabolic bone disease or abnormal PTH level were excluded. Data from banked serum and routinely collected structured questionnaire were used. Serum samples were collected and archived according to the time of collection in boxes containing 100 samples, and stored at –80°C. The cases of low BMD were defined as children with speed of sound under the 20th percentile, while the controls of standard BMD were defined as being greater than or equal to the 20th percentile according to the common standard separated by gender and age [4]. The cases were randomly selected from the screening population by using a computerized random number function, with the specific box of the cases identified. Controls were then sourced by using the remaining samples in each box. The full box was analyzed by the trained laboratory technicians without knowing the all subjects' case or control status. As a result, 4,846 samples consented to participate in the study. After written informed consent was obtained, a face to face investigation was conducted by the prepared doctors with the structured questionnaire to collect related details including gender, age, weight, height, gestational age and season, delivery mode, birth weight, feeding patterns, milk intake during pregnancy and for children, VD or calcium supplementation during pregnancy and for children, time of outdoor activity during pregnancy and for children, and so on.

### Bone ultrasound and serum 25(OH)D measurement

The BMD were determined by using the quantitative ultrasound BMD scanner (Sunlight Omnisense TM 7000, Israel). The trained doctors measured SOS at the left mid-tibia of children and repeated once again. Serum samples were thawed once, and serum 25(OH)D concentrations was determined by using an *in vitro* diagnostic enzyme immunoassay kit OCTEIA 25-Hydroxy Vitamin D (Immunodiagnostic Systems, Boldon, United Kingdom) according to the instructions. The variations between intra- and inter-assay coefficients were less than 9.0%, and the reported analytic sensitivity of the immunoassay was 6.8–380 nmol/L. Commonly used cutoffs to define 25(OH)D status were assigned at 50 and 75 nmol/L [4, 15, 16]. More than 10% results were selected to repeat for quality control at random.

### Statistical analysis

The 25(OH)D concentrations and BMD by the selected characteristics were described as mean ± SD, and differences were calculated by the Student's *t*-test or One-Way ANOVA. Differences of the low BMD prevalence by 25(OH)D concentrations and selected characteristics were calculated by χ^2^ test. As the distribution of serum 25(OH)D concentrations was obviously skewed toward the left, we converted it to the Log10 [Lg25(OH)D] for the next analysis. The associations of 25(OH)D concentrations with BMD and stakes of low BMD were estimated by using multiple linear regression and logistic regression analyses, respectively, in which the key potential confounders were taken into account. The relation between 25(OH)D and BMD, and between 25(OH)D and stakes of low BMD were explored by a smoothing plot. The relation between 25(OH)D and BMD was analyzed by using Pearson's test. All the statistical analyses were performed with R software (version 2.13.0), and *P* ≤ 0.05 in a two-sided test was interpreted as statistically significant.
